# Risk Prescriptions of Strong Opioids in the Treatment of Chronic Non-Cancer Pain by Primary Care Physicians in Catalonia: Opicat Padris Project

**DOI:** 10.3390/ijerph19031652

**Published:** 2022-01-31

**Authors:** Aina Perelló-Bratescu, Christian Dürsteler, Maria Asunción Álvarez-Carrera, Laura Granés, Belchin Kostov, Antoni Sisó-Almirall

**Affiliations:** 1 Larrard Primary Health Center, Parc Sanitari Pere Virgili, 08024 Barcelona, Spain; aperello@perevirgili.cat; 2 Primary Healthcare Transversal Research Group, IDIBAPS, 08036 Barcelona, Spain; badriyan@clinic.cat; 3 Pain Medicine Section, Anaesthesiology Department, Hospital Clínic de Barcelona, 08036 Barcelona, Spain; dursteler@clinic.cat; 4 Surgery Department, Medicine Faculty, Universitat de Barcelona, 08036 Barcelona, Spain; 5 Pharmacy Service, Parc Sanitari Pere Virgili, 08023 Barcelona, Spain; aalvarez@perevirgili.cat; 6 Preventive Medicine and Epidemiology Department, Hospital Clinic Barcelona, 08036 Barcelona, Spain; granes@clinic.cat; 7 Primary Care Centre Les Corts, Consorci d’Atenció Primària de Salut Barcelona Esquerra (CAPSBE), 08028 Barcelona, Spain; 8 Medicine Department, Medicine Faculty, Universitat de Barcelona, 08036 Barcelona, Spain

**Keywords:** analgesics, opioids, opioid-related disorders, inappropriate prescribing, chronic pain, physicians, primary care, drug combinations, big data, pharmacoepidemiology

## Abstract

The prescription of strong opioids (SO) for chronic non-cancer pain (CNCP) is steadily increasing. This entails a high risk of adverse effects, a risk that increases with the concomitant prescription of SO with central nervous system depressant drugs and with the use of SO for non-recommended indications. In order to examine this concomitant risk prescription, we designed a descriptive, longitudinal, retrospective population-based study. Patients aged ≥15 years with a continued SO prescription for ≥3 months during 2013–2017 for CNCP were included. Of these, patients who had received concomitant prescriptions of SO and risk drugs (gabapentinoids, benzodiazepines and antidepressants) and those who had received immediate-release fentanyl (IRF) were selected. The study included 22,691 patients; 20,354 (89.7%) patients received concomitant risk prescriptions. Men and subjects with a higher socioeconomic status received fewer concomitant risk prescriptions. Benzodiazepines or Z-drugs were prescribed concomitantly with SO in 15,883 (70%) patients, antidepressants in 14,932 (65%) and gabapentinoids in 11,267 (49%), while 483 (21.32%) patients received IRF (2266 prescriptions in total) without a baseline SO. In conclusion, our study shows that a high percentage of patients prescribed SO for CNCP received concomitant prescriptions with known risks, as well as IRF for unauthorized indications.

## 1. Introduction

Chronic pain worsens the quality of life, mental health and the cardiovascular risk, and increases health spending [1]. It is one of the main reasons for consultation in primary care, and pain-relieving drugs are the most frequently consumed [2,3]. These include strong opioids (SO), whose consumption has increased in recent years in our setting [4,5], mostly in patients with chronic non-cancer pain (CNCP). This indication lacks evidence and has a high risk of adverse effects, leading guidelines to recommend limiting their use to very specific cases, after assessing the risk-benefit [6,7,8,9]. The risk increases with some factors such as age, comorbidity and concomitant prescription of some other drugs.

Among these drugs, the most common are benzodiazepines. Several guidelines advise against their association with SO due to the risk of respiratory depression [7,10], increased mortality and hospitalizations [11,12], and an increased risk of SO overdoses [13], especially in older people taking high doses of SO. Despite this evidence, a recent survey revealed that a high percentage of primary care physicians still prescribe SO in association with benzodiazepines [14]

Likewise, cases of serotonergic syndrome have been described when SO are associated with antidepressants and antipsychotics, especially tapentadol [15,16].

The concomitant prescription of gabapentinoids with SO has been associated with increased mortality [17], especially with moderate and high doses of gabapentin [18] and high doses of pregabalin [19], which double the risk of mortality from SO overdoses.

Transmucosal immediate-release fentanyl (IRF) formulations present a high risk of adverse effects such as abuse or addiction [10], with the only authorized indication in cancer patients suffering breakthrough pain and undergoing baseline SO treatment [20]. Despite this, IRF is still prescribed off-label in a high percentage of cases [10,14,21].

The reasons for this high worldwide SO prescription are multifactorial, and the socio-economic consequences are significant. In the US, opioid consumption has been described as an epidemic (183,000 deaths between 1999 and 2015) [22]. Organizations such as the CDC have warned of its risks and recommended reducing their prescription [23]. In the EU, even though the situation is not yet so alarming, there is significant evidence concerning both the health risks and the lack of evidence for the benefits of SO prescription in CNCP [5,10,24,25,26]. In the Spanish health system, the main reasons for this are a disproportionate expectation by the general population of drug effectiveness and the lack of access to non-pharmacological options of proven efficacy [27,28], as discussed later.

The aim of this study was to examine potentially dangerous concomitant prescription of SO with other drugs acting on the central nervous system by primary care physicians over a 5-year period, as well as off-label IRF prescriptions. Our study shows that a high percentage of patients prescribed SO for CNCP received concomitant prescriptions with known risks, as well as IRF for unauthorized indications.

## 2. Materials and Methods

### 2.1. Design

We carried out a longitudinal, retrospective, descriptive study using data obtained through the Data Analysis Program for Health Research and Innovation (PADRIS). The PADRIS mission is to make health data available to promote research, innovation and evaluation in health through access to the reuse and cross-over of health data generated by the public health system of Catalonia (SISCAT), in accordance with the legal and regulatory framework, ethical principles and transparency towards citizens of the program. It collects detailed individual-level information on demographics and socioeconomic characteristics, and exhaustive health-related and medical resource use information generated by interactions between users and the public healthcare system that provides universal public health coverage to all Catalan residents. It permits epidemiological analyzes, evaluations of healthcare interventions and programs, and public analysis and benchmarking of health indicators across healthcare areas, among other assessments. Specifically, for healthcare-related data, the database has, since 2012, progressively collected information from several sources, including the Minimum Basic Dataset for Healthcare Units registry (which includes hospitalizations, primary care visits, and skilled nursing facility visits), information on pharmacy prescription fillings, and billing records, including outpatient specialist visits and emergency department visits. The register includes an automated validation system, and regular external audits are carried out. The data obtained were anonymized, eliminating potentially identifiable records.

The data request was made in December 2018. Previously, an agreement was signed for the transfer of anonymized health data between AQUAS (Agència de Qualitat i Avaluació Sanitàries de Catalunya), responsible for the PADRIS program, and IDIBAPS (Institut d’Investigacions Biomèdiques August Pi i Sunyer), which covers the research activity of several authors of this study. The first database was provided by AQUAS in March 2019 and the final database, after clarifying some issues regarding the data request, was received in February 2020.

### 2.2. Study Population

The PADRIS program was asked to select patients with following inclusion criteria: Cat Salut (Catalan Public Health Coverage) users aged ≥15 years (when Spanish patients move from being attended by pediatricians to primary care physicians), in whom a primary care physician had prescribed an SO for CNCP for ≥3 months continuously in the previous five years (2013–2017). The duration of the SO prescription for ≥3 months was based on the definition of chronic pain in the current guidelines as pain that persists or recurs for >3 months [2,29]. A list of oncological diagnoses was provided to exclude patients with cancer in the last 5 years. Patients receiving parenteral opioids (except transdermal opioids) were also excluded, as they are not usually used for CNCP.

### 2.3. Variables Included

- Concomitant risk prescription: defined as the concomitant prescription of SO with any of the active ingredients included in the following pharmacological groups (central nervous system depressant drugs and concomitant drugs with adverse effects proven): antidepressants [15,16], gabapentinoids [13,17,18,19], benzodiazepines and Z-drugs [7,10,11,12]. For extraction they were identified by their Anatomical Therapeutic Chemical Classification (ACT) code.
- The prescription of any of these drugs during the 100 days after the date of prescription of the SO was considered a concomitant prescription (according to chronic pain definition as ≥3 months).
- Doses of pregabalin and gabapentin prescribed in patients with a concomitant prescription of SO (variable included due to dose-related mortality in gabapentinoids concomitancy [18,19]). The dose per unit (tablet, capsule) was obtained and the daily dose was calculated using the dosage interval recommended in the data sheet (gabapentin every 8 h, pregabalin every 12 h).
- Prescription of IRF without prescription of a baseline SO in the previous 100 days (unauthorized prescription [20]).

### 2.4. Ethical Aspects

The procedures followed Spanish and Catalan laws. Researchers followed the ethical standards of the Declaration of Helsinki for biomedical studies and the activities described followed the Code of Good Practice in clinical research.

The data were anonymized, and the study protocol was presented and approved by the Research Ethics Committee of the Hospital Clinic of Barcelona (Ref. HCB/2018/0749).

### 2.5. Statistical Analysis

Categorical variables are presented as absolute frequencies and percentages and continuous variables as the mean and standard deviation (SD). The Chi-square test was used to study the association between concomitant medication and sex, socioeconomic level, age and health region. All significance tests were two-tailed and values of *p* < 0.05 were considered significant. The statistical analysis was made using R version 3.6.1 for Windows.

## 3. Results

We included 22,691 patients (0.29% of the total insured population in 2017) who had received SO for ≥3 months for CNCP (without a cancer diagnosis) in 2013–2017 prescribed by primary care physicians in Catalonia (Spain): 15,389 (67.7%) patients were aged >70 years and 17,509 (77.2%) were female.

Concomitant risk prescriptions were received by 20,354 (89.7%) patients. Comparison of patients receiving risk prescriptions with those who did not (Table 1) shows that males and subjects with a higher socioeconomic status received fewer concomitant risk prescriptions than females and people with lower incomes (*p* < 0.001). Prescription of concomitant risk prescriptions decreased with older age.

### 3.1. Association between Strong Opioids (SO) and Benzodiazepines

**Figure 1:**
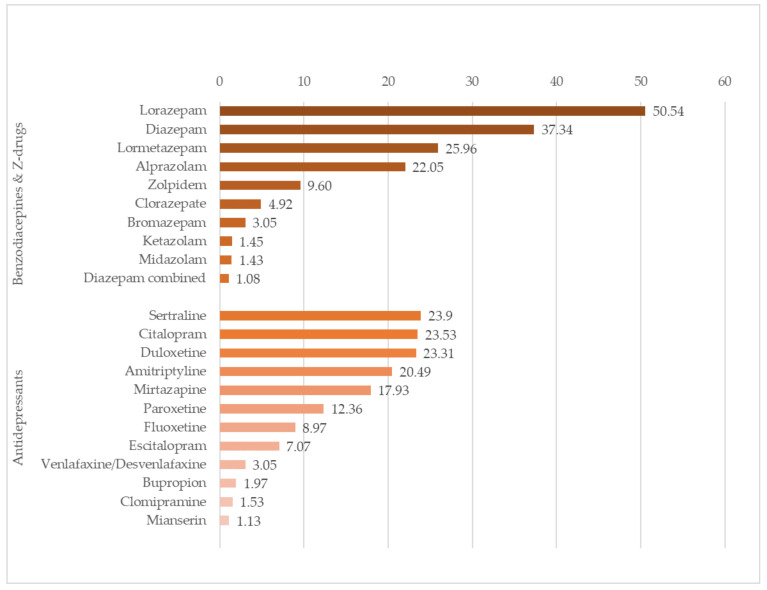
Types of benzodiazepines, Z-drugs and antidepressants concomitantly prescribed with strong opioids (SO). Percentage of each drug among the total patients receiving concomitant prescriptions, by pharmacological group.

Benzodiazepines or Z-drugs were prescribed concomitantly with SO in 15,883 (70%) patients, of whom 9084 (57%) were aged >75 years, and 12,800 (80.6%) were female. Table 2 shows the types of benzodiazepine prescribed. The most frequently prescribed was lorazepam, in more than half the patients (Figure 1).

### 3.2. Association between SO and Antidepressants

Concomitant prescriptions of antidepressants and SO were received by 14,932 (65%) patients, of whom 8287 (55.5%) were aged >75 years and 12,205 (81.7%) were female. Table 3 shows the type of antidepressants and the frequency of their prescription. The most common were selective serotonin reuptake inhibitors (SSRIs) in 11,388 (76.2%) prescriptions.

### 3.3. Association between SO and Gabapentinoids

Concomitant prescriptions of SO and gabapentinoids were received by 11,267 (49%) patients (Table 4), of whom 47.81% were aged ≥75 years and 74% were female. The daily doses of gabapentin prescribed were moderate (900–1799 mg) in 72.46% of prescriptions and high (≥1800 mg) in 21.36%. For pregabalin, 26.36% of prescriptions were for doses of >300 mg daily.

The distribution of the type of SO prescribed concomitantly is similar in all three combination modalities. Transdermal fentanyl was prescribed in 55–60% cases, followed by tapentadol, in 27–31% of cases, and oxycodone in 24–29% of cases.

IRF was received by 483 (21.32%) patients (2266 total) without a baseline SO; 4275 prescriptions (15% of total) of IRF (28,488) were without a baseline SO.

## 4. Discussion

This study is the first to examine risk prescriptions of SO for CNCP made by primary care physicians in Catalonia during a 5-year period, using big data analysis. The vast majority of patients received concomitant risk prescriptions, especially young women with low incomes. We found a high percentage of concomitant prescription of SO and benzodiazepines, antidepressants and gabapentinoids, of which a quarter were prescribed at high doses. Nearly a quarter of IRF prescriptions were indicated without a baseline SO.

Almost 90% of the population studied received concomitant risk prescriptions. A few reports have analyzed the concomitant prescription of SO with gabapentinoids and benzodiazepines. Vold et al. [30] described 50% of patients receiving SO who also received benzodiazepines and Z drugs, and 11% gabapentinoids. Musich et al. [31] found that 15–28% of patients had concomitant prescriptions of SO and central nervous system (CNS) depressants. Torrance et al. [17] found that, of patients receiving gabapentinoids, 60% had concomitant prescriptions for SO and/or benzodiazepines.

We found concomitant prescription was higher in females with lower incomes, and in younger patients. Some studies described a higher consumption of SO in persons with lower incomes, such as that by Friedman et al. [32], or the concomitant prescription of benzodiazepines and SO found by Sharma et al. [11]. Torrance et al. [17] found a higher concomitant prescription of gabapentinoids in women with lower incomes, as we did, but also in older people, which we did not. Sharma et al. described the same concomitant prescription profile as Torrance, in this case the association of benzodiazepines and SO. In any case, a progressive increase in concomitant prescriptions was observed with age, until a decrease was observed from 85 years onwards. Approximately half the patients receiving concomitant prescriptions were aged >75 years. This is especially worrying, since the risk of adverse effects of concomitant prescribing increases with age, as seen in the studies by Puustinen et al. [33] and Musich et al. [31], which found that the concomitant prescription of SO with medications that affect the CNS increased the risk of cognitive decline and falls/fractures.

We observed concomitant prescription of benzodiazepines and Z-drugs with SO in 70% of patients, a higher proportion than in other studies. Sharma et al. [11] found a rate of 24% of concomitant prescriptions while Zin et al. [34] found a rate of 12%, with the risk of adverse effects of concomitant prescription being related to the dose and duration of the SO intake, which was higher in older patients. The high concomitant prescription of benzodiazepines in our sample is troubling since, in several studies, such as those by Macleod et al. [13] and Yang et al. [12], there is evidence of an increased risk of death from SO overdose in patients with a concomitant prescription of benzodiazepines. The same effect is analyzed by other studies, such as the recent study by Liu et al. [35]. The risk of death was not associated with a dose-dependent effect of Z-drugs. Nurminen et al. [36] described an increased risk of fracture in men aged >65 years with a concomitant prescription of benzodiazepines and SO.

Sixty-five percent of our sample received a concomitant prescription of antidepressants and SO. Among the most common antidepressants were SSRIs, as well as duloxetine and amitriptyline, which are also used for neuropathic pain, and mirtazapine, used more frequently to treat insomnia. It is logical to think that patients with CNCP could also have depression and insomnia, justifying these associations. Khan et al. [37] found an association between antidepressant use and a higher risk of SO consumption, and at higher doses. On the other hand, the combination of SO with antidepressants increases the risk of serotonergic syndrome, described with the use of fentanyl [38] or in several alerts with tapentadol [4,15].

Nearly half the patients in our study were prescribed concomitantly with gabapentinoids, mostly pregabalin (more than a quarter at high doses). The use of these drugs in concomitant risk prescriptions has become widespread in recent years as Yu warned [39], with an increase in deaths related to SO in patients with concomitant prescription of gabapentinoids, as demonstrated by Torrance et al. [17]. Gomes et al. [18] showed an increase in deaths related to SO of 60% with moderate (900–1799 mg daily) and high (≥1800 mg daily) gabapentin doses combined with SO (virtually all patients in our study received gabapentin doses of >900 mg/day). Another study by the same author [19] found a 68% increase in deaths related to SO in patients who received pregabalin concomitantly, especially at high doses (>300 mg daily). A study by Nielsen et al. [40] related the use of pregabalin with the consumption of high doses of SO.

Nearly a quarter of patients receiving IRF for CNCP in our study did not have a baseline SO treatment. IRF has a high economic impact, but above all a high risk of adverse effects such as abuse and addiction [10]. In addition, it is only authorized in cancer patients with breakthrough pain receiving a baseline treatment with SO [10,41]. All patients in our cohort received off-label IRF, as they are not cancer patients. This problem in our setting was described by González-Bermejo et al. [21], who found a prevalence of 25% in patients with IRF for CNCP.

The limitations of the study include the fact that it was based on the date of prescription of concomitant drugs, and we assumed that SO were prescribed for a minimum of 3 months as selected chronic treatments. In some isolated cases, the SO could be interrupted before 3 months and not coincide with the risk drug, although the joint prescription was made by primary care physicians, who usually prescribe this type of drugs for a minimum of 3 months.

In summary, the causes of these high percentages of risk prescriptions are multifactorial. The inadequate training that physicians receive in pain in our setting, as shown by several studies [14,42] and the limitation in access to non-pharmacological treatments with proven efficacy such as physiotherapy or psychology [27,28] may have decisively influenced the results of this study.

One proposal to decrease risk prescriptions in the short-term would be to add alerts to computerized medical records when prescribing SO with another central nervous system depressant or IRF without a baseline SO or in patients suffering non-cancer pain. Recently, the Spanish health ministry has included mandatory validation for IRF prescriptions, which will help reduce risk prescriptions.

In any case, physicians should identify patients with concomitant risk prescriptions and closely monitor possible adverse effects, ideally in conjunction with their reference pain unit.

## 5. Conclusions

A high percentage of patients prescribed SO for CNCP received concomitant prescriptions with known risks (benzodiazepines, antidepressants and gabapentinoids), as well as IRF for unauthorized indications. Men and subjects with a higher socioeconomic status received fewer concomitant risk prescriptions.

## Figures and Tables

**Table 1:** Patients with and without concomitant medication.

	No Concomitant Medication (*n* = 2337) (10.2%)	Concomitant Medication (*n* = 20,354) (89.7%)	*p*-Value
	***n*** **(%)**	***n*** **(%)**	
Sex			<0.001
Female	1594 (9.1)	15,915 (90.9)	
Male	743 (14.3)	4439 (85.7)	
Socioeconomic level (in Euros)			<0.001
Exempt	107 (7.8)	1261 (92.2)	
<18,000	1910 (10.4)	16,478 (89.6)	
18,001–100,000	316 (10.9)	2592 (89.1)	
>100,000	4 (14.8)	23 (85.2)	
Age (in years)			<0.001
<50	129 (7.1)	1683 (92.9)	
50–64	287 (7.3)	3661 (92.7)	
65–74	340 (9.1)	3388 (90.9)	
75–84	660 (10.4)	5697 (89.6)	
85–94	750 (12.7)	5143 (87.3)	
≥ 95	171 (17.9)	782 (82.1)	
Health region			0.317
Urban	1490 (10.3)	12,962 (89.7)	
Semi-urban	568 (10.2)	4989 (89.8)	
Rural	279 (10.4)	2400 (89.6)	

**Table 2:** Benzodiazepines and Z-drugs concomitantly prescribed with strong opioids.

Benzodiazepines	No. Patients	%
Lorazepam	8027	50.54
Diazepam	5931	37.34
Lormetazepam	4124	25.96
Alprazolam	3502	22.05
Zolpidem	1525	9.6
Clorazepate	782	4.92
Bromazepam	485	3.05
Ketazolam	231	1.45
Midazolam	227	1.43
Diazepam in combination with pyridoxine and/or sulpiride	172	1.08

**Table 3:** Antidepressants concomitantly prescribed with SO.

Antidepressants	*n* Patients	%
Sertraline	3569	23.9
Citalopram	3514	23.53
Duloxetine	3480	23.31
Amitriptyline	3060	20.49
Mirtazapine	2678	17.93
Paroxetine	1845	12.36
Fluoxetine	1340	8.97
Escitalopram	1056	7.07
Venlafaxine/Desvenlafaxine	456	3.05
Bupropion	294	1.97
Clomipramine	228	1.53
Mianserin	169	1.13

**Table 4:** Gabapentinoids in concomitant prescription with SO.

Gabapentinoids	No. Patients	%
Gabapentin	5573	49.46
Pregabalin	7564	67.13

## Data Availability

The data from the PADRIS program are not available to public.
